# Cross‐Feeding of Carbon and Nitrogen Between Aquificales and *Thermus* in Hot Springs

**DOI:** 10.1111/1462-2920.70225

**Published:** 2026-01-07

**Authors:** Lisa M. Keller, Daniel R. Colman, Andri Stefánsson, Eric S. Boyd

**Affiliations:** ^1^ Department of Microbiology and Cell Biology Montana State University Bozeman Montana USA; ^2^ Institute of Earth Sciences University of Iceland Reykjavik Iceland

**Keywords:** carbon dioxide fixation, chemoautotrophy, chemosynthesis, nitrogen fixation, reverse TCA cycle, syntrophy

## Abstract

Acquisition and cycling of carbon and nitrogen among members of hot spring communities are not well understood. Metagenomic analyses of 105 communities inhabiting high temperature hot springs across Yellowstone and Iceland showed a co‐distribution pattern of putatively autotrophic and/or diazotrophic (nitrogen‐fixing) Aquificales and *Thermus* populations. Targeted enrichment of autotrophic and diazotrophic populations in an Icelandic hot spring produced a co‐culture of *Pampinifervens* (Aquificales) that encoded carbon dioxide and nitrogen fixation pathways and *Thermus* (Thermales). Growth experiments revealed *Pampinifervens* could support the fixed carbon and nitrogen demands of *Thermus*, enabling growth. Interestingly, growth of *Thermus* was enhanced in co‐cultures when *Pampinifervens* was forced to fix both carbon and nitrogen versus just carbon (ammonia‐amended cultures). Further experimentation with *Thermus*, when grown in isolation, showed it preferred amino acids over ammonia as a nitrogen source. These findings demonstrate the importance of metabolic interactions among populations that can dictate the co‐distribution of taxa in hot springs, drive community assembly, and maintain biodiversity. Further, these results highlight the fundamental role of Aquificales in the functioning of hot spring ecosystems, particularly those limited in organic carbon and fixed nitrogen like those in Iceland and to a lesser extent Yellowstone.

## Graphical abstract

*Pampinifervens* and *Thermus* populations are co‐distributed in hot springs in Yellowstone and Iceland. Genome reconstruction suggests *Pampinifervens* Flu05 cross feeds *Thermus* organic carbon and nitrogen, a phenomenon that was verified via co‐culture studies. These observations provide insight into the functional interactions that drive the assembly of hot spring microbial communities.

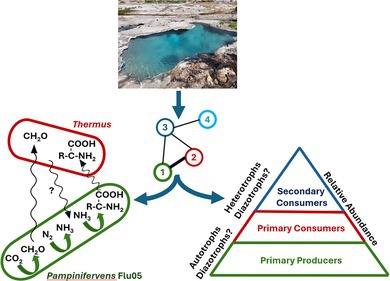

## 1 Introduction

Members of the bacterial order Aquificales are common in hydrothermal vent systems and high temperature hot spring communities, where they are often dominant primary producers (Eder and Huber 2002; Meyer‐Dombard et al. 2005; Huber and Eder 2006; Reysenbach et al. 2006; Reysenbach et al. 2009; Meyer‐Dombard et al. 2011; Hou et al. 2013; Takacs‐Vesbach et al. 2013; Zeng et al. 2021; Colman et al. 2024) due to the exclusion of photoautotrophic organisms (Cox et al. 2011; Boyd et al. 2012). Dominant Aquificales in terrestrial hot springs include those affiliated with the genera *Hydrogenobaculum*, *Hydrogenobacter*, *Sulfurihydrogenibium*, *Thermocrinis*, and a newly described genus, *Pampinifervens* (Meyer‐Dombard et al. 2005; Reysenbach et al. 2006; Meyer‐Dombard et al. 2011; Hou et al. 2013; Takacs‐Vesbach et al. 2013; Palmer et al. 2025). Members of Aquificales tend to be microaerophiles that utilise hydrogen (H_2_), thioarsenite, and dissolved sulfide (H_2_S (aq) + HS^−^), and its oxidation products such as thiosulfate (S_2_ $O_{3}^{2-}$) and elemental sulfur (S^0^), to support chemolithoautotrophic metabolism (Huber et al. 1998; Takai et al. 2003; Aguiar et al. 2004; Nakagawa et al. 2005; Reysenbach et al. 2006; Caldwell et al. 2010; Dodsworth et al. 2015), although some strains are facultative anaerobes (Kawasumi et al. 1984; Suzuki et al. 2001; Takai et al. 2001; Takai et al. 2003; Aguiar et al. 2004; Nakagawa et al. 2005; Huber and Eder 2006).

**FIGURE 1:**
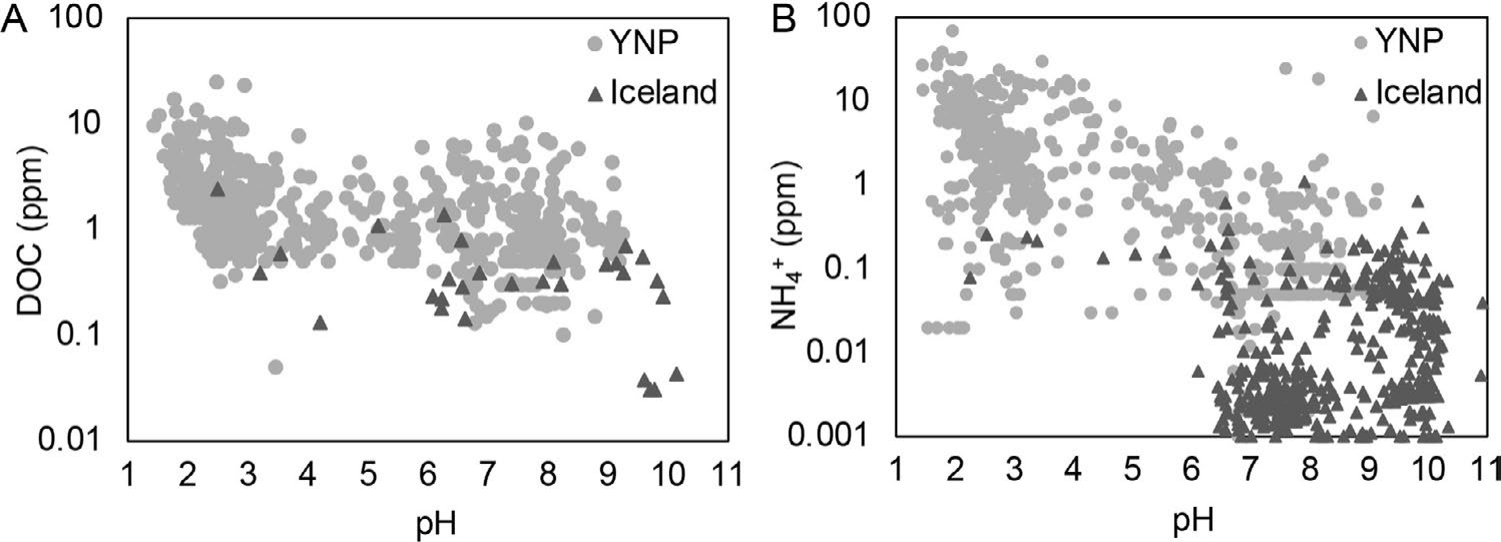
Dissolved organic carbon (DOC; A) and ammonia/ammonium (NH_3_/N$H_{4}^{+}$; B) concentrations in Yellowstone National Park (YNP) and Icelandic hot spring waters. DOC and NH_3_/N$H_{4}^{+}$ concentrations were compiled from previously published data (Stefánsson 2017; McCleskey et al. 2022) from each geographic location and are plotted on a log scale as a function of spring pH.

Chemolithoautotrophic aquificales are particularly prevalent in circumneutral to alkaline hot springs where they can form filamentous streamers in outflow channels (Havig et al. 2011; Meyer‐Dombard et al. 2011; Takacs‐Vesbach et al. 2013). Consistent with their inferred role as primary producers, circumneutral to alkaline springs tend to be sourced by deep hydrothermal aquifers that have low dissolved organic carbon (DOC) content (McCleskey et al. 2005; Stefánsson 2017; Nye et al. 2020; McCleskey et al. 2022) (Figure 1A). This is attributed to these springs being hydrologically isolated from surface input (Sims et al. 2023), a predominant source of DOC in surface‐influenced acidic springs (Nye et al. 2020). Likewise, circumneutral to alkaline hot springs tend to have limited availability of ammonia (NH_3_)/ammonium (N$H_{4}^{+}$) (Holloway et al. 2011; Ásgeirsdóttir 2016), a feature that is attributed, at least in part, to volatilization of N$H_{3}^{0}$_(g)_ at neutral pH (pK_a_ of N$H_{4}^{+}$_(aq)_ ↔ N$H_{3}^{0}$_(g)_ = 6.2 at 100°C (Amend and Shock 2001)) (Figure 1B) (Stefánsson 2017; McCleskey et al. 2022). The limited availability of DOC and ammonia in these springs would be expected to select for autotrophic organisms with the capability to fix atmospheric dinitrogen (N_2_) during microbial community assembly. Autotrophic, N_2_‐fixing (i.e., diazotrophic) members of the Aquificales have previously been isolated from or detected via molecular methods in hot springs (Eder and Huber 2002; Hamilton et al. 2014; Nishihara et al. 2018; Colman et al. 2024; Palmer et al. 2025). This includes putatively autotrophic and diazotrophic *Thermocrinis* (Eder and Huber 2002; Hamilton et al. 2014), *Hydrogenobacter* (Nishihara et al. 2018), *Hydrogenobaculum* (Colman et al. 2024), and *Pampinifervens* (Palmer et al. 2025). Further, carbon and nitrogen isotopic data measured in biomass collected from circumneutral to alkaline springs, including Aquificales‐dominated filaments, is suggestive of CO_2_ and N_2_ fixation in some springs (Macko and Estep 1984; Havig et al. 2011).

Aquificales often dominate hot springs that range in pH from below 3 to above 9 and are typically found alongside heterotrophic organisms (Power et al. 2018; Colman et al. 2024). In circumneutral to alkaline hot springs, heterotrophic organisms consistently include those associated with the bacterial genus *Thermus* and the archaeal genus *Pyrobaculum* (Meyer‐Dombard et al. 2011; Fernandes‐Martins et al. 2021; Fernandes‐Martins et al. 2023; Keller et al. 2023). For example, analysis of 13 circumneutral to alkaline hot springs in Yellowstone National Park (YNP) consistently showed the co‐occurrence of dominant populations of *Thermocrinis* and subdominant populations of *Thermus* (Keller et al. 2023). Similarly, in high‐temperature hot springs in New Zealand and China, Aquificales are often the dominant population alongside subdominant *Thermus* (Power et al. 2018; Guo et al. 2021; Sriaporn et al. 2023). *Thermus* are typically considered heterotrophic (Brock and Freeze 1969; Williams and da Costa 1992), which may explain their common co‐occurrence with autotrophic members of Aquificales in DOC‐limited springs. However, more recent studies identified *Thermus* strains that encode the Calvin Cycle, including *Thermus scotoductus* and several other *Thermus* sp. (Müller et al. 2016; Fernandes‐Martins et al. 2023; Colman et al. 2024). Nevertheless, cultivation studies conducted to date have yet to equivocally demonstrate autotrophic growth in *T. scotoductus* or other *Thermus* strains (Skirnisdottir et al. 2001). To our knowledge, *Thermus* strains capable of fixing N_2_ have not been described to date. Thus, existing data suggest *Thermus* strains are either fully or partially dependent on exogenous sources of fixed carbon and/or nitrogen.

**FIGURE 2:**
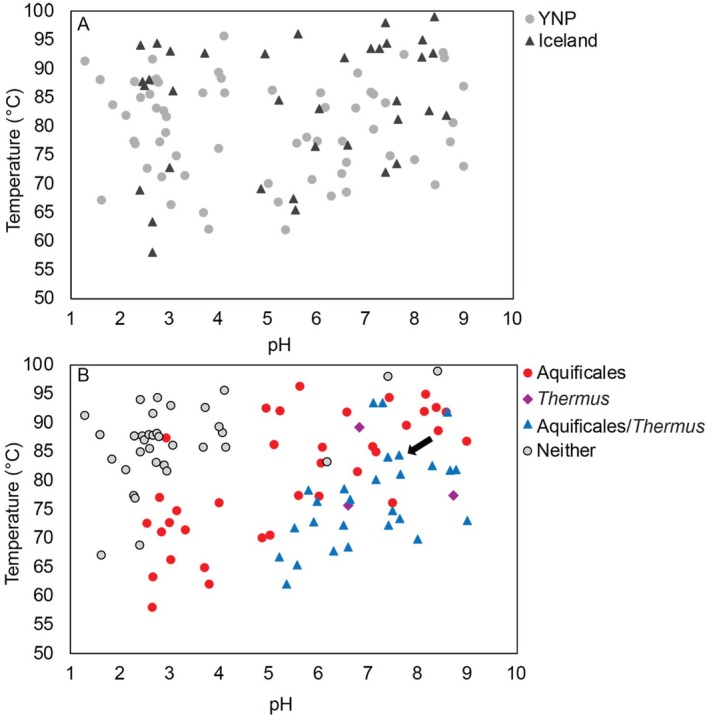
Temperature and pH of hot springs in Yellowstone National Park (YNP) and Iceland analysed in this study (A), and an overlay of the distribution of Aquificales‐ and *Thermus*‐affiliated metagenome assembled genomes (MAGs) in hot spring sediment and water column communities (B). Metagenomes were generated from genomic DNA from sediments or the water columns of 58 different YNP hot springs (grey circles) and 36 different Icelandic hot springs (dark grey triangles) that together span a range of pH (1.29–9.0) and temperature (58°C–99°C) (A). Metagenomes were analysed for MAGs affiliated with the Aquificales (red circles), *Thermus* (purple diamonds), or both Aquificales and *Thermus* within the same spring sample (blue triangles) (B). Grey circles depict hot springs in YNP or Iceland where community metagenomes lacked MAGs affiliated with both Aquificales and *Thermus*. The black arrow highlights Flu05, the Icelandic hot spring from where a *Pampinifervens* (Aquificales) and *Thermus* co‐culture was generated.

Based on previous results discussed above, it was hypothesized that the strong pattern in the co‐distribution of Aquificales and *Thermus* is, at least in part, due to nutrient limitation, specifically fixed nitrogen and carbon (Figure 1A,B), that selects for autotrophic members of the Aquificales that cross‐feed fixed carbon and/or nitrogen species to co‐inhabiting *Thermus* strains. Secondarily, the more extreme fixed nitrogen limitation in Iceland springs than Yellowstone springs (Figure 1B) was hypothesized to necessitate a greater interdependency of *Thermus* on Aquificales metabolites and that this would be reflected in patterns in their co‐distribution. To assess these interrelated hypotheses, the distribution, diversity, and function of Aquificales and *Thermus* in water and/or sediments from 105 communities from YNP (58 springs, several sampled multiple times) and Iceland (36 springs) was examined (Figure 2). Enrichment assays from a spring in Iceland produced a co‐culture of *Pampinifervens* and *Thermus* that was then used to begin to examine the nature of interactions between these populations.

## 2 Materials and Methods

### 2.1 Sample Collection and Geochemical Analysis

Sediment and/or planktonic samples were collected from 36 Icelandic hot springs using methods previously described (Colman et al. 2024). Briefly, surface sediments (top several mm) were collected from springs using flame‐sterilised spatulas or sampling cups in late summer of 2022, transferred to sterile bottles, and stored on ice (for use in cultivation) or frozen in the field using dry ice. Planktonic samples for metagenomic sequencing were collected as previously described (Keller et al. 2023). Sediment collected for cultivation was placed in 70 mL serum bottles and the bottles were filled completely with spring water to create a slurry. Bottles were then capped with sterile rubber stoppers, sealed with aluminium crimp caps, and stored on ice or in a refrigerator (4°C) until cultures were inoculated. Spring pH and temperature were measured using a WTW combination pH probe (Weilheim, Germany) and conductivity was measured using a temperature‐compensated YSI meter (YSI Inc., Yellow Springs, OH) (Table S1). Dissolved oxygen (DO) was measured using a PSt3 oxygen dipping probe attached to a Fibox 4 DO instrument (PreSens, Regensburg, Germany) in the water column of each spring sampled as previously described (Keller et al. 2025). Select geochemical and sequence data previously reported from 58 YNP hot springs (several of which were sampled multiple times over several years) were included in the study (Colman et al. 2024) (Table S1). Similarly, geochemical data from a variety of YNP spring (McCleskey et al. 2022) and Icelandic spring and well (Stefánsson 2017) waters were included in the study, where indicated.

### 2.2 DNA Extraction and Metagenomic and Analysis

Genomic DNA was extracted from sediments as previously described (Colman et al. 2024). Briefly, duplicate extractions were performed on 0.5–1.0 g subsamples of sediment using the FAST DNA Spin Kit for soil (MP Biomedicals, Irvine, CA) according to manufacturer guidelines. Following extraction and purification, DNA was quantified with the Qubit dsDNA high sensitivity assay kit and fluorometer (Invitrogen, Waltham, MA). Equal volumes of duplicate extracts were pooled to generate a minimum of 10 ng of DNA for metagenomic library preparation and sequencing at the Genomics Core Facility at the University of Wisconsin‐Madison. Libraries were sequenced with the Illumina NovaSeq S4 platform (2 × 150 bp paired‐end reads) to a depth of ~70–100 million quality‐filtered reads each. Reads were processed and analysed using the same workflow as previously described (Colman et al. 2024). MAGs that were affiliated with the bacterial order Aquificales and genus *Thermus* were identified based on GTDB taxonomic classifications and were compiled (Table S2). MAGs were then screened for genes encoding carbon fixation and nitrogen fixation (molybdenum‐nitrogenase) pathways, as described previously (Fernandes‐Martins et al. 2021). Briefly, the Kyoto Encyclopedia for Genes and Genomes (KEGG) database and the METABOLIC software program (version 4.0) (Zhou et al. 2022) were used to identify homologues of proteins involved in carbon fixation pathways (rTCA cycle in Aquificales MAGs, Calvin cycle in *Thermus* MAGs) and genes encoding the structural components of nitrogenase (NifHDK) (Boyd et al. 2011). A table of MAGs that encode N_2_ or CO_2_ fixation pathways can be found in Table S3. Average nucleotide identity (ANI) was calculated to compare MAGs affiliated with the same genus using the fastANI program (v.1.32) (Jain et al. 2018).

### 2.3 Phylogenomic Analysis and Functional Annotations of Aquificales‐ and Thermus‐Affiliated MAGs

Genomes and MAGs from representatives within the order Aquificales and the genus *Thermus* were obtained from the NCBI database. MAGs that were identified taxonomically to be members of the bacterial order Aquificales or the genus *Thermus* from the 69 YNP and 36 Iceland metagenomes were compiled and included in the analysis (Table S2). The markerfinder script (https://github.com/faylward/markerfinder) was then used to search the genomes for 30 universal marker genes that were then aligned with ClustalO (v.1.2.4) (Sievers et al. 2011) and concatenated. The concatenated alignment was subjected to Maximum‐Likelihood phylogenetic reconstruction using IQ‐Tree (Nguyen et al. 2014). Model finder plus (MFP) was used to determine the best substitution model for the dataset and 1000 ‘ultrafast’ bootstraps were used to determine branch support. Outgroups were included in the phylogenetic analyses, and the phylogeny was visualised and edited using the Interactive Tree of Life (ITOL) program (Letunic and Bork 2021).

### 2.4 Enrichment Culturing, Sequencing, and Analysis

Sediments from Flúðir spring 5 (Flu05; 64.13697 N, 20.30981 W) located in the town of Flúðir, Iceland, were selected for cultivation‐based enrichment of Aquificales and *Thermus*. Mineral salts enrichment medium contained (g L^−1^): CaCl_2_ • 2H_2_O (0.01), KCl (0.33 g), MgCl_2_ • 6H_2_O (0.33), and Na_2_SO_4_ • 10H_2_O (0.03). The pH of the medium was adjusted to 7.0 using NaOH. All glassware used in experiments was soaked in trace metal grade 1 M hydrochloric acid (HCl) overnight and then rinsed in MilliQ water. Thirty millilitre of media was dispensed into 70 mL serum bottles, and these were capped and sealed prior to autoclave sterilisation. After autoclaving and while serum bottles were still hot, filter‐sterilised phosphate buffer (pH 7.0) was added to a final concentration of 10 mM. Bottles were then sparged for 20 min with N_2_ passed over heated (250°C) and hydrogen (H_2_)‐reduced copper shavings. The headspace (40 mL) of serum bottles was adjusted to a final composition of (vol./vol.): 58% N_2_, 20% carbon dioxide (CO_2_), 20% hydrogen (H_2_), and 2% oxygen (O_2_) unless otherwise noted. Anoxic and filter‐sterilised solutions of SL‐10 trace metals (Widdel et al. 1983) and Wolfe's Vitamins (Atlas 1996) were added using N_2_‐flushed syringes and needles to final concentrations of 2 mL L^−1^. A serial dilution (10‐fold) approach with 3.0 mL of the sediment‐spring water slurry (~100 mg of sediment) as inoculum was used. Enrichment cultures were incubated on their sides at 80°C on a platform shaking incubator (50 rpm) to increase infusion of headspace gases into the medium. Enrichment progress was monitored by enumeration of cells, as previously described (Keller et al. 2025). Briefly, 2 μL of 4′,6‐diamidino‐2‐phenylindole (DAPI; 2 μg/mL final concentration) was added to 0.5 mL sub‐samples of culture that were then incubated at room temperature (~21°C) for 15 min. Samples were treated with detergent [100 mM ethylenediaminetetraacetic acid (EDTA), 100 mM sodium pyrophosphate, 1% (v/v) Tween 80] to disperse cells, filtered onto 0.22 μm black polycarbonate filters (Millipore, Billerica, MA), and enumerated using an Evos fluorescent microscope (Life Technologies, Carlsbad, CA). After three transfers of the most dilute culture, cells were harvested for genomic sequencing. Thirty millilitre of a log phase culture were harvested via centrifugation (4696×*g*, 30 min, 4°C) to pellet cells. The cell pellet was then subject to DNA extraction and quantification as described above. Genomic DNA from the enrichment culture was sequenced via the Illumina NovaSeq platform at Microbial Whole Genome Sequencing (Pittsburgh, PA, USA). Sequence data were assembled, annotated, and analysed as previously described (Keller et al. 2025), resulting in three bins (Table S4). The proteins encoded in the two most abundant bins (described below), as predicted with the program METABOLIC, are reported in Table S7.

This first round of enrichment culturing produced the co‐culture containing two populations of *Pampinifervens* and one population of *Thermus*. This co‐culture was used to evaluate cross‐feeding behaviours between these populations. Attempts to isolate the dominant *Pampinifervens* from the secondary *Pampinifervens* and *Thermus* populations were unsuccessful and resulted in the isolation of the Bin 3 *Pampinifervens* population (described below). The Bin 3 *Pampinifervens* was isolated through further rounds of dilution to extinction transfers (5 transfers), where cells were transferred in the early stages of growth (less than 24 h) under N_2_‐fixing, autotrophic conditions.

To isolate *Thermus* from the co‐culture, Brock 1978/1981 *Thermus* medium (Williams and da Costa 1992) was used with slight modifications. The medium contained (g L^−1^): nitrilotriacetic acid (NTA) (0.1), CaSO_4_ • 2H_2_O (0.06), MgSO_4_ • 7H_2_O (0.1), KNO_3_ (0.103), and NaNO_3_ (0.689). The pH was adjusted to 7.0 and 10 mL of media were distributed into Balch tubes, which were then sealed and capped prior to autoclaving. After autoclaving and prior to inoculating, medium was supplemented with Wolfe's vitamins and SL‐10 as described above and the headspace was left as air with no adjustments. A 1 mL (10% v/v) inoculum of the original co‐culture grown under N_2_‐fixing conditions was utilized and cells were given acetate (5 mM final concentration) as the sole carbon and energy source. The bottles were incubated at 70°C until they reached turbidity (~2.5 days) and were then used to inoculate serial dilutions (10‐fold) out to 10^−3^. This dilution series was repeated twice. After two rounds of dilution, the 10^−3^ culture was subject to DNA extraction using the same methods described above. PCR with *Pampinifervens* Flu05‐ or *Thermus*‐specific primers (Table S5), described in detail below, were used to confirm isolation of *Thermus* from the *Pampinifervens* populations. Once isolation was confirmed, experiments using the isolated *Thermus* were performed.

### 2.5 Nutrient Cross‐Feeding Experiments

Mineral salts medium, prepared as described above, was used in all nutrient cross‐feeding cultivation experiments. Fifty‐five millilitre of media was dispensed into 165 mL serum vials that were then sealed, autoclaved, and purged as described above. The final headspace composition for N_2_ cross‐feeding experiments was (vol./vol.): 58% N_2_, 20% CO_2_, 20% H_2_, and 2% O_2_. Wolfe's vitamin solution and SL‐10 trace metal solution were added prior to inoculating as described above. In non‐N_2_ fixing experiments, ammonium chloride (NH_4_Cl, 0.33 g L^−1^) was added as a source of fixed nitrogen and the final headspace composition was adjusted to: 78% H_2_, 20% CO_2_, and 2% O_2_. The inoculum for all cross‐feeding experiments was grown under N_2_/CO_2_ fixing conditions with no organic nitrogen or organic carbon source provided. Experiments were incubated on their sides at 80°C in a platform shaking incubator (50 rpm). Growth was monitored using a combination of cell counts (described above) and quantitative PCR (qPCR) of genes encoding the 16S rRNA gene region using primers specific for *Pampinifervens* Flu05 and *Thermus*.

To generate enough DNA for qPCR assays in N_2_‐fixing cultures, triplicate serum bottles were inoculated, and these were harvested at 6, 12, and 24 h. In the case of non‐N_2_ fixing cultures, small aliquots (2 mL) were sufficient to recover enough DNA for qPCR assays between 12 and 48 h of incubation. Triplicate (sub)samples were harvested at each timepoint via centrifugation (4696×*g*, 30 min, 4°C). Cell pellets were subject to DNA extraction and purification as described above. DNA was subject to qPCR using methods previously described (Keller et al. 2025). Briefly, primers were designed to specifically amplify a portion of the 16S rRNA gene from either *Pampinifervens* Flu05 or *Thermus* using the NCBI Primer BLAST tool. Although there were two populations of *Pampinifervens* in the enrichment culture (Bin 1 and Bin 3, Table S4), the completeness for Bin 3 was low and did not include a 16S rRNA sequence. As such, primers for *Pampinifervens* Flu05 (Bin 1) and *Thermus* (Bin 2) were designed, and the annealing temperature was optimised through gradient PCR. Primer specificity was tested using DNA extracted from the co‐culture and DNA extracted from a pure culture of *Pampinifervens* (Bin 3). Gel products were screened for any non‐specific amplification and band size was evaluated to verify that primers were amplifying the desired products (Figure S5). Primer sequences and annealing temperatures are reported in Table S5.

Once primers were optimised and checked for specificity, qPCR was conducted using the SYBR Green Supermix (Bio‐Rad, Hercules, CA) in a final volume of 20 μL. One μL of genomic DNA containing a known amount of DNA (see above) and 0.5 μL of both the forward and reverse primers (0.5 mM final concentration) were added to each reaction and reactions were subject to the following cycling conditions in the CFX Connect Real‐time system (Bio‐Rad): Initial denaturing at 95°C for 1 min, followed by 35 cycles of 95°C for 30 s, annealing at specified temperature (Table S5) for 1 min, and extension at 72°C for 30 s, with a plate read step at the end of each cycle. This was followed by a melt curve from 65°C to 95°C with 0.5°C increases every 5 s. Plasmid standards for converting threshold amplification signals to gene copy number were prepared as previously described (Keller et al. 2025).

### 2.6 *Thermus* Isolate Growth Experiments

Experiments involving the isolated *Thermus* strain were performed using mineral salts medium used for initial enrichment culturing (not the Brock medium) and cross‐feeding experiments. Thirty millilitre of media was dispensed into 70 mL and prepared as described above. To determine the impact of nitrogen source on growth, triplicate cultures were either provided with NH_4_Cl, casamino acids, or glutamic acid to a final concentration of about 6 mM. Casamino acids were added at the same weight per volume ratio as glutamic acid since they do not have a defined molecular mass. Cultures were grown aerobically and were provided with acetate (5 mM final concentration) as a carbon and energy source. Bottles were incubated at 70°C in a static incubator. Cell counts were performed as described above. To determine whether *Thermus* cells were able to grow on their own under cross‐feeding conditions, bottles were prepared as described for the cross‐feeding experiments with a nitrogen fixing condition (CO_2_/N_2_) and non‐nitrogen fixing conditions (CO_2_/N$H_{4}^{+}$). Additionally, a positive control grown with NH_4_Cl and acetate under an aerobic headspace and a negative control grown with no provided nitrogen or carbon source were included. To limit carryover of fixed carbon and nitrogen, inoculum was washed with base media. Growth was monitored via cell counts as described above.

## 3 Results and Discussion

### 3.1 Co‐Distribution of Aquificales and *Thermus*

**FIGURE 3:**
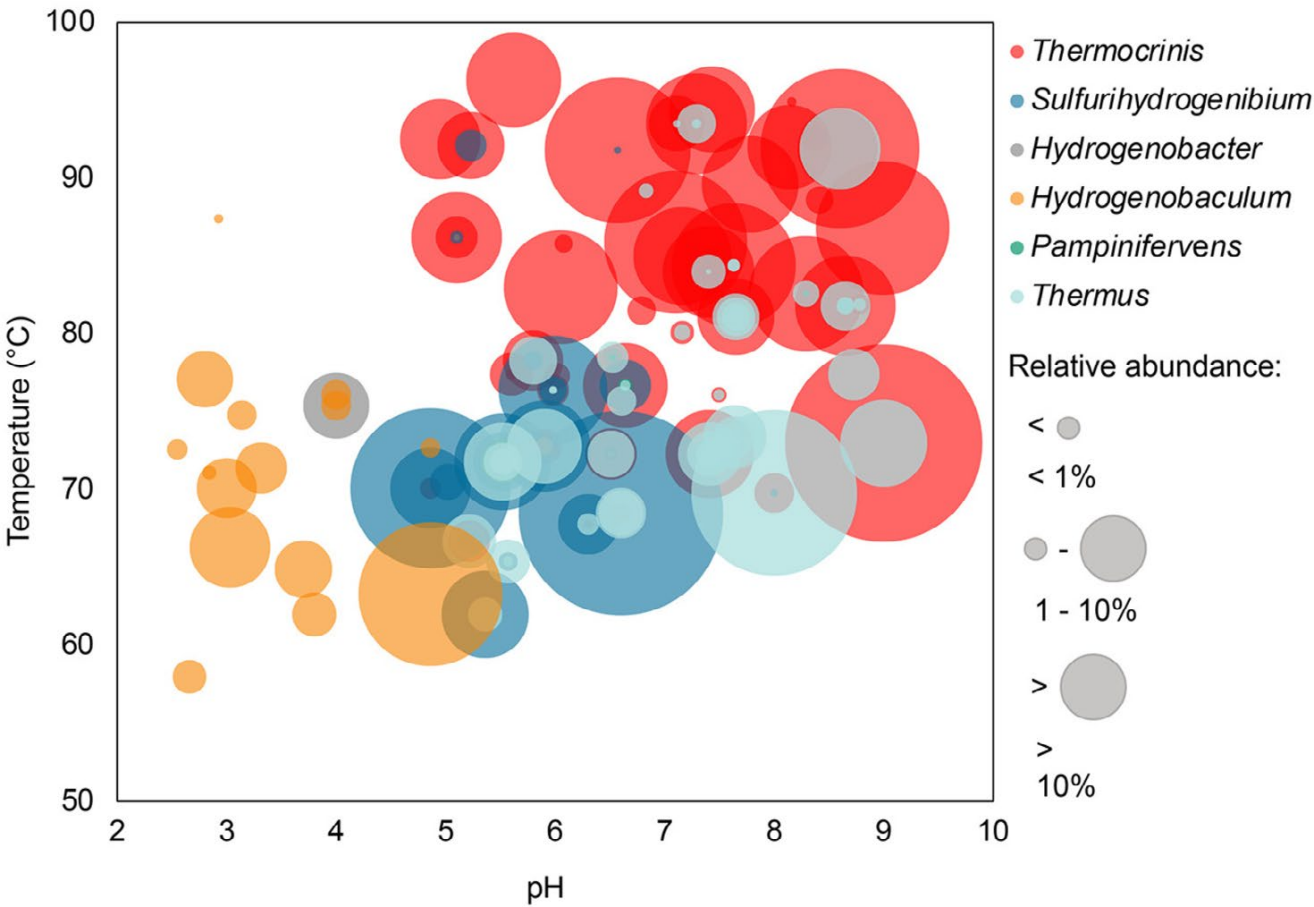
The distribution and relative abundance of Aquificales‐ and *Thermus*‐affiliated metagenome‐assembled genomes (MAGs) recovered from 105 Yellowstone and Iceland hot spring microbial communities. Each bubble represents a MAG affiliated with a member of the order Aquificales or genus *Thermus* and is plotted as a function of the pH and temperature of the hot spring that it was recovered from. The size of the bubble corresponds to the relative abundance of that MAG (% of binned reads) in the hot spring planktonic or sediment community and the colour corresponds to the genus. Plots showing the individual pH, temperature, and abundance distributions of MAGs from each spring can be found in Figure S1.

Metagenomic and 16S rRNA gene amplicon sequence data from previous studies reveal a strong pattern in the co‐distribution of populations affiliated with the bacterial order Aquificales and the bacterial genus *Thermus* in the water column (i.e., planktonic) and sediment communities from circumneutral to alkaline hot springs globally (Meyer‐Dombard et al. 2011; Power et al. 2018; Fernandes‐Martins et al. 2021; Guo et al. 2021; Colman et al. 2023; Keller et al. 2023; Sriaporn et al. 2023; Colman et al. 2024). To gain a better understanding of a potential functional relationship underpinning this pattern of co‐distribution, metagenomes from 69 planktonic and sediment communities from hot springs in YNP (Lindsay et al. 2018; Colman et al. 2019; Payne et al. 2019; Colman et al. 2020; Fernandes‐Martins et al. 2021; Fernandes‐Martins et al. 2023; Keller et al. 2023; Sims et al. 2023; Colman et al. 2024; Fernandes‐Martins et al. 2024) and 36 communities from hot springs in Iceland were screened for MAGs affiliated with Aquificales and *Thermus* (Table S1). The sampled hot springs ranged in pH from 1.3 to 9.0 and temperature from 58°C to 99°C (Figure 2A). Of the 105 communities examined, 65 contained MAGs affiliated with the order Aquificales, including those affiliated with the genera *Hydrogenobaculum*, *Sulfurihydrogenibium*, *Hydrogenobacter*, *Thermocrinis*, and *Pampinifervens*. Aquificales were present in springs ranging in pH from 2.6 to 9.0 and temperature from 58°C to 96°C (Figure 2B). The distribution of Aquificales genera was pH‐ and temperature‐dependent, with *Hydrogenobaculum*‐affiliated MAGs predominating in lower temperature (< 80°C) acidic (pH < 5.0) springs, *Sulfurihydrogenibium*‐ and *Hydrogenobacter*‐affiliated MAGs predominating in lower temperature (< 80°C) moderately acidic to neutral (pH 5.0–7.0) springs, and *Thermocrinis*‐affiliated MAGs predominating in higher temperature (70°C–96°C) moderately acidic to alkaline springs (pH > 5.0–9.0) (Figures 3 and S1). This is consistent with previous reports of the distribution of Aquificales genera in hot springs (Reysenbach et al. 2006; Hou et al. 2013; Takacs‐Vesbach et al. 2013; Colman et al. 2024). MAGs affiliated with *Pampinifervens* were identified in springs that ranged in pH from 5.5 to 8.7 and in temperature from 65.4°C to 82.6°C (Figure 3 and S1) and exhibited < 70% (range of 51%–69%) AAI with other Aquificales MAGs, consistent with the previous conclusion that they represent a distinct genus of Aquificales (Palmer et al. 2025). *Thermocrinis*‐, *Sulfurihydrogenibium‐*, and *Hydrogenobaculum‐*affiliated MAGs were often dominant among hot spring communities, with a maximum abundance of 77.0%, 82.6%, and 40.9% of binned communities, respectively (Figures 3 and S1). The abundance of these populations as determined by the percent of mapped reads, which incorporates both binned and unbinned sequences for each metagenome, is also reported for all MAGs analysed for this dataset (Table S2). In contrast, *Hydrogenobacter‐* and *Pampinifervens*‐affiliated MAGs were present at much lower relative abundances, making up a maximum of 8.7% and 3.6% of binned communities, respectively (Figures 3 and S1).

MAGs affiliated with *Thermus* were identified in 34 of the 105 metagenomes, and these spanned a pH range of 5.5–9.0 and temperature range of 62°C–94°C (Figure 2B; Figures 3 and S1). This is consistent with the pH and temperature range of springs where *Thermus* has been previously identified (Brock and Freeze 1969; Williams and da Costa 1992; Chung et al. 2000; Skirnisdottir et al. 2001; Albuquerque et al. 2014). *Thermus‐* and Aquificales‐affiliated MAGs co‐occurred in 29 of the 34 springs that contained *Thermus*, with only three springs that contained *Thermus* not containing a member of Aquificales (Figure 2B). One of the samples that contained *Thermus* but not Aquificales was a sediment sample from Roadside West Spring (RSWS), sampled and analysed in a previous study (Fernandes‐Martins et al. 2023). While the RSWS sediment community did not include Aquificales‐affiliated MAGs, the planktonic community was comprised of over 80% Aquificales‐affiliated MAGs. Aquificales are often dependent on O_2_ for energy generation and therefore tend to be more dominant in planktonic communities relative to sediment communities (Hou et al. 2013; Colman et al. 2016; Fernandes‐Martins et al. 2021). This is thought to be due to the limited solubility of O_2_ at high temperature, resulting in limited transport into deeper regions of springs where sediments are located (Amend and Shock 2001; Fernandes‐Martins et al. 2021). Indeed, dissolved O_2_ concentrations decreased from 96.9 μM in the water column of RSWS to 12.5 μM at a depth of only ~0.5 cm into the sediment layer (Keller et al. 2025). This suggests that the perceived absence of certain populations may be due to sampling only sediment or water for a given spring.

**FIGURE 4:**
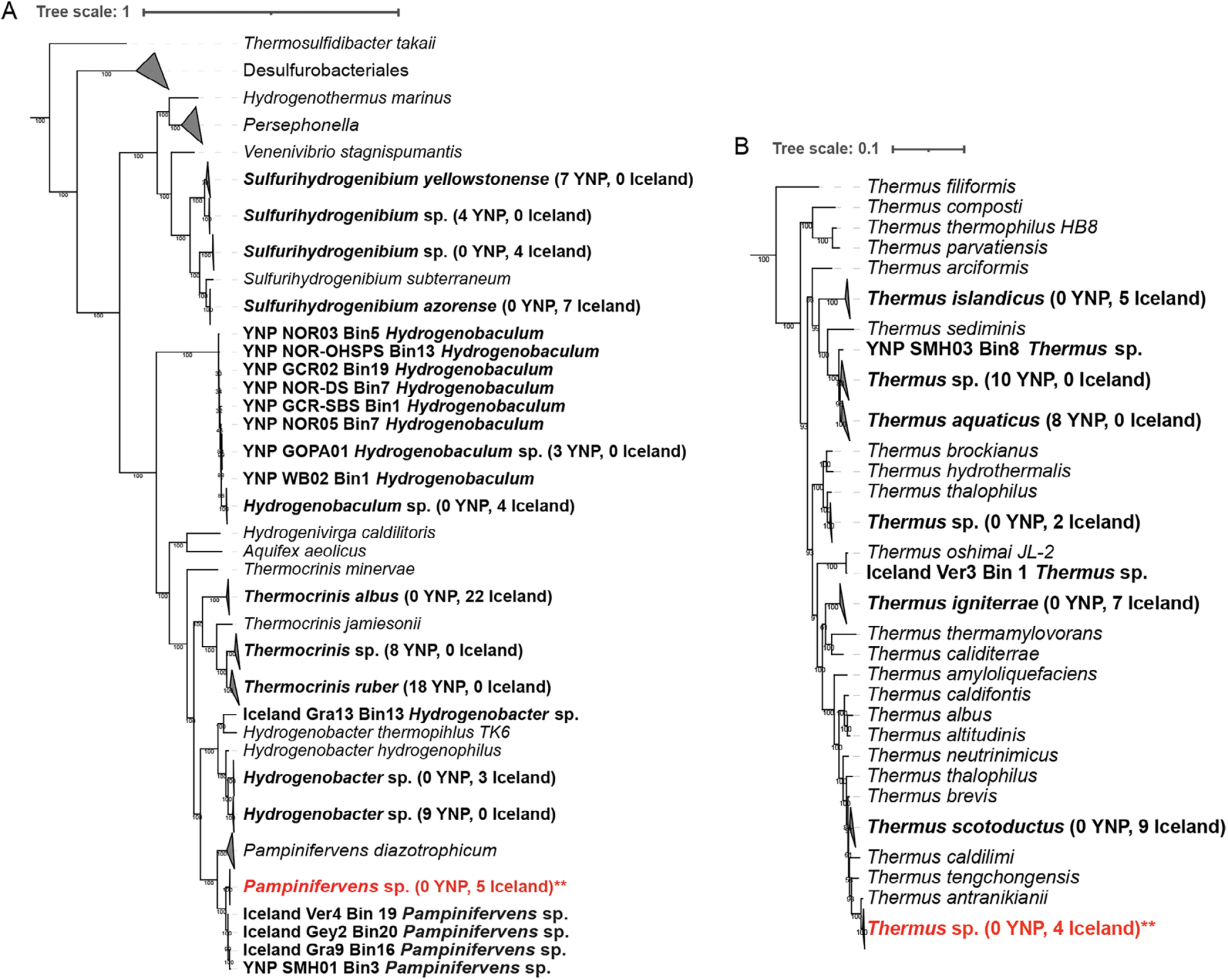
Phylogenomic position of Aquificales‐ and *Thermus*‐affiliated metagenome assembled genomes (MAGs). Reference genomes for cultured isolates and MAGs collected from 94 hot springs in Yellowstone National Park and Iceland were used to build phylogenomic trees of organisms affiliated with the order Aquificales (A) and the genus *Thermus* (B). Bolded names depict MAGs generated in this study. Location of the spring from which the MAG is from is indicated in the name. ** and red font represents the organisms identified in the co‐culture discussed in Figures 6, 7, 8.

Despite consistency in the pH and temperature range where Aquificales and *Thermus* were identified in YNP and in Iceland (Figure 2B), MAGs from each location were phylogenetically distinct, with MAGs of individual genera or species from Yellowstone forming clades that were distinct from those of Iceland (Figure 4). The sole exception to this was *Pampinifervens*, where the single MAG identified in YNP (in a spring metagenome from the Seven Mile Hole location) grouped with those from Iceland. The general pattern of distinct Aquificales in YNP versus Iceland is consistent with a recent study that found 16S rRNA gene evidence indicating strain‐ to species‐level endemism in Aquificales from YNP and Iceland, likely driven by geochemical differences between the two locations (Colman et al. 2023). Similarly, Power et al. 2024 identified an Aquificales population (*Venenivibrio* sp.) in New Zealand hot springs that has yet to be identified in other hot springs globally, suggesting endemism of that population to New Zealand (Power et al. 2024). ANI values for Aquificales‐ and *Thermus*‐affiliated MAGs generated from comparisons within region (i.e., YNP vs. YNP and Iceland vs. Iceland) were higher than ANI values generated from between region comparisons (i.e., YNP vs. Iceland) for each of the genera analysed (Table S6). Although there is little variation among clades of the same genera within regions for Aquificales, the distinct grouping and lower ANI values when comparing YNP to Iceland MAGs suggests differentiation based on region. Dispersal limitation of *Sulfolobus* from globally distributed hydrothermal systems was previously inferred to allow local evolution to occur, leading to global strain‐level divergences (Whitaker et al. 2003). However, recent high‐throughput comparative analysis of hot spring communities in Iceland, YNP, and Japan strongly indicated that geochemical variation attributable to differences in the bedrock hosting the springs and/or the different tectonic regimes is involved in differentiation of hot spring microorganisms (Colman et al. 2023).

### 3.2 Geochemical Differences Between YNP and Iceland Hot Springs

YNP and Iceland hot springs are hosted in two distinct geologic and tectonic settings, with the YNP hydrothermal system attributed to a shallow mantle plume hosted mainly in silica‐rich, rhyolitic bedrock (Christiansen 2001; Huang et al. 2015) and the Iceland hydrothermal system attributed to a mantle hot spot at an oceanic spreading center hosted in primarily iron‐rich, basaltic bedrock (Arnórsson 1995). Geologic or tectonic settings have been suggested to influence microbial communities due to differences in temperature and volatiles associated with each type of system (Fullerton et al. 2021; Colman et al. 2023; Upin et al. 2023).

To gain a better understanding of differences in DOC and dissolved ammonia concentrations between YNP and Iceland hot springs, previously published data from each system were compiled (Stefánsson 2017; McCleskey et al. 2022). DOC concentrations in YNP hot springs ranged from below detectable limits to 24.8 ppm (0–2.1 mM), with acidic springs containing on average more DOC than alkaline hot springs (Figure 1A), consistent with previous analyses of DOC in these springs (McCleskey et al. 2022). In Iceland hot springs, DOC was far lower (below detection to 2.4 ppm, 0–200 μM) and was not enriched in either acidic or alkaline hot springs (Figure 1A). Dissolved ammonia concentrations in YNP springs ranged from below detection to 69.1 ppm (0–4.6 mM), with acidic springs on average containing higher concentrations of dissolved ammonia compared to alkaline springs (Figure 1B). The disparity in dissolved ammonia between acidic and alkaline springs is likely due to a combination of volatilization of N$H_{3}^{0}$_(g)_ and decompressional boiling of fluids as they ascend to the surface (Fournier 1989; Holloway et al. 2011; Hamilton et al. 2014). While circumneutral to alkaline springs are volatile poor (e.g., low dissolved ammonia), acidic springs tend to be enriched in volatiles due to condensation of gases with near surface fluids (Hurwitz and Lowenstern 2014). Interestingly, Icelandic springs have significantly less dissolved ammonia (0–1.09 ppm; 0–70 μM) than those in YNP (Figure 1B). This difference may be attributable to the bedrock hosting the hydrothermal systems: Rhyolites that primarily host hot springs in YNP contain higher concentrations of nitrogen compared to basalts that primarily host hot springs in Iceland (Holloway and Dahlgren 2002).

### 3.3 Metabolic Differences Between Aquificales‐ and *Thermus‐*Affiliated MAGs in YNP and Iceland Hot Springs

**FIGURE 5:**
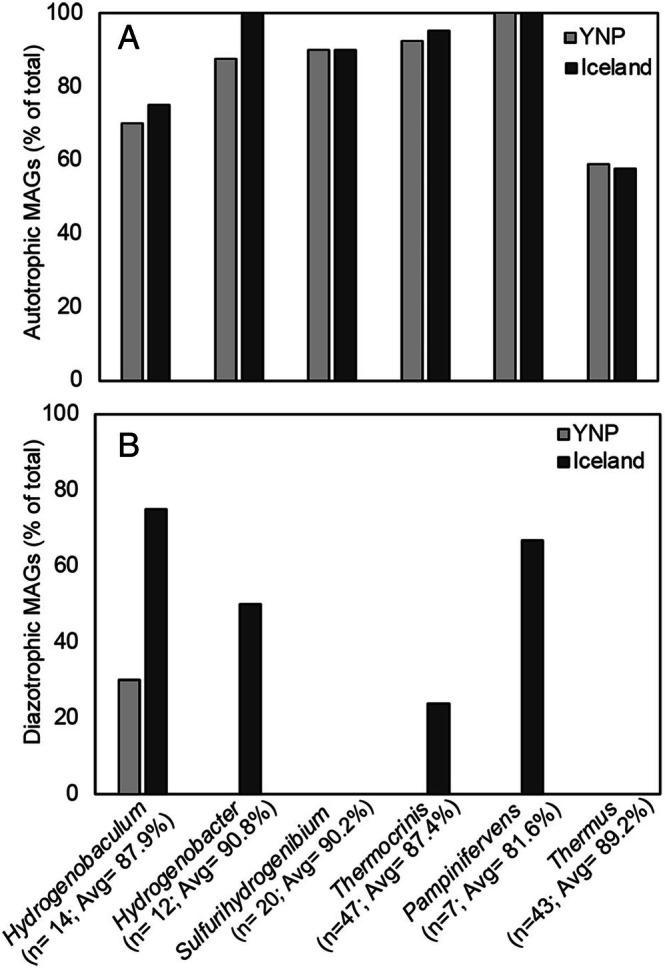
Distribution of metagenome‐assembled genomes (MAGs) affiliated with specified Aquificales genera and *Thermus* that encode pathways for autotrophic (A) and/or nitrogen‐fixing (diazotrophic) metabolisms (B) in Yellowstone National Park (YNP) and Iceland hot spring microbial communities. MAGs affiliated with five genera of Aquificales and the genus *Thermus* were detected in the metagenomic dataset, and these were analysed for the presence of protein encoding genes involved in CO_2_ fixation (rTCA and Calvin cycles) and nitrogen fixation (molybdenum nitrogenase, NifHDK). The number of MAGs identified for each genus (n) is depicted, as is the average (Avg) completeness for all the MAGs analysed for each genus.

Although alkaline springs in YNP have low concentrations of DOC and dissolved ammonia compared to the acidic springs, they are still higher than in Icelandic springs (Figure 1A). Such differences could affect the distribution of autotrophic and N_2_‐fixing Aquificales and *Thermus* and the nature of their interactions. Aquificales‐ and *Thermus*‐affiliated MAGs were therefore examined for their autotrophic and N_2_‐fixing potentials. Regardless of geographic location, > 70% of Aquificales MAGs and > 50% of *Thermus* MAGs encoded necessary genes for the rTCA (ATP citrate lyase or alternative citrate cleavage mechanism) and Calvin cycles (ribulose‐1,5‐bisphosphate) of CO_2_ fixation, respectively, with no apparent difference between locations (Figure 5A). The absence of genes encoding CO_2_‐fixation pathways in remaining Aquificales MAGs is attributed primarily to incompleteness of the MAGs, with those that lacked key marker genes being on average 81.2% complete (with a minimum of 51.8% and maximum of 98.1%). While CO_2_ fixation is not generally considered to be widespread in *Thermus*, MAGs were on average 89.2% complete and could therefore also be an underestimate of the percentage of strains within this genus that are capable of CO_2_ fixation. As such, the availability of DOC and the differences in DOC between hydrothermal systems is apparently not a strong driver of the distribution of putatively autotrophic Aquificales and *Thermus*. Interestingly, phylogenetic positioning also does not seem to be a strong driver of whether *Thermus* populations encode the necessary genes for CO_2_ fixation (Figure S2; Table S3). The observation that > 50% of *Thermus*‐affiliated MAGs encoded CO_2_ fixation pathways (Figure 5A) was surprising given that this genus has historically been described as heterotrophic. However, several recent studies have suggested the genetic potential for autotrophy via the Calvin cycle in *Thermus* based on sequencing data (Müller et al. 2016; Fernandes‐Martins et al. 2023; Colman et al. 2024). Additional physiological experiments are needed to determine if Calvin cycle‐encoding *Thermus* are capable of autotrophic growth or if they are mixotrophic (described in more detail below).

Aquificales‐ and *Thermus*‐affiliated MAGs were also screened for homologues of genes encoding the structural components of molybdenum nitrogenase (NifHDK; Nif), since all N_2_‐fixing organisms encode Nif, and only a subset of Nif‐encoding genomes also encode alternative nitrogenases (Boyd et al. 2011). No *Thermus*‐affiliated MAG encoded homologues of Nif, regardless of geographic location. In contrast, Nif homologues were identified among Aquificales‐affiliated MAGs from both locations, with a marked difference in the taxonomic distribution and abundance of Nif‐encoding MAGs between locations (Figure 5B). In YNP, the only Aquificales‐affiliated MAGs that encoded Nif were affiliated with *Hydrogenobaculum*, and these MAGs were identified in only a single acidic spring but were detected in two separate years [spring pH was 2.6 and 4.9 in each of those years (Colman et al. 2024)]. Interestingly, acidic springs like these, which are influenced by input of volcanic gases (Fournier 1989), tend to have higher concentrations of dissolved ammonia than circumneutral to alkaline springs (Figure 1B) (Holloway et al. 2011; Hamilton et al. 2014). Surprisingly, minimal evidence for N_2_‐fixing Aquificales (or other N_2_‐fixing Archaea or Bacteria) was identified in MAGs from higher pH springs [(Figure 5B); (Colman et al. 2024)]. In contrast, in Iceland hot springs, Aquificales‐affiliated MAGs that encoded Nif were widespread, with MAGs affiliated with *Sulfurihydrogenibium* being the only genera of Aquificales that did not encode Nif (Figure 5B). Among Iceland Aquificales MAGs, 75% of *Hydrogenobaculum* MAGs, 50% of *Hydrogenobacter* MAGs, 24% of *Thermocrinis* MAGs, and 67% of *Pampinifervens* MAGs encoded Nif homologues (Figure 5B). It is possible, if not likely, that the enrichment in N_2_‐fixing Aquificales in Iceland is due to lower concentrations of dissolved ammonia in hot spring waters compared to YNP. Indeed, *Thermocrinis albus* , isolated from a circumneutral spring in Iceland (Eder and Huber 2002), encodes Nif (Boyd and Peters 2013), whereas *Thermocrinis ruber* , isolated from a circumneutral to alkaline spring in YNP, lacks homologues of Nif. Although an Aquificales strain with the ability to fix N_2_ has yet to be isolated from YNP, PCR‐based studies of the distribution of genes encoding the nitrogenase iron protein (NifH) indicated that the potential for N_2_ fixation among Aquificales exists in circumneutral to alkaline YNP springs (Hamilton et al. 2011; Hamilton et al. 2014). Previous studies have also detected nitrogenase encoding sequences in unbinned YNP metagenomic data that likely belonged to a member in the order Aquificales (Colman et al. 2024). Together, these data indicate that there are potentially N_2_‐fixing Aquificales populations in YNP that were not captured in this study due to data limitations. Regardless, these observations suggest that N_2_‐fixing Aquificales are less abundant in YNP than in Iceland hot springs.

### 3.4 Co‐Culturing of *Pampinifervens* and *Thermus* and Metabolic Analysis

It was hypothesized that the co‐distribution patterns of Aquificales and *Thermus* in circumneutral to alkaline springs in YNP and Iceland was due to cross feeding of fixed carbon and/or fixed nitrogen, especially in Iceland. To better understand the nature of the putative interaction(s) between Aquificales and *Thermus*, enrichment cultures targeting autotrophic, N_2_‐fixing organisms were initiated with samples from Iceland with the goal of producing a co‐culture of a member of the Aquificales and *Thermus*. Sediments from the Flu05 hot spring sample (Figure S3) were used to inoculate enrichment medium. At the time of sampling, Flu05 had a temperature of 84.4°C and a pH of 7.6 (Figure 2B, black arrow is depicting Flu05) and was chosen due to likelihood of containing both Aquificales and *Thermus* based on the temperature and pH distributions where these organisms commonly co‐occur (Figure 2B) and the presence of streamers in the outflow channel (Figure S3). Flu05 contained abundant dissolved hydrogen (3.1 μM), sulfide (18.8 μM), and oxygen (3.3 μM), suggesting this to be an environment conducive to (micro)aerobic H_2_‐ or sulfide‐oxidising and autotrophic Aquificales.

Initial enrichments were set up with mineral medium and a headspace comprising the following gases (vol./vol.): 58% N_2_, 20% CO_2_, 20% H_2_, and 2% O_2_. Enrichments were incubated statically in the dark at 80°C, slightly lower than the temperature of Flu05. After several rounds of dilution to extinction transfers, a single rod morphotype, similar to that of *Thermocrinis* and *Thermus*, was identified and DNA was extracted to sequence and identify the organism(s). Three MAGs were identified in the gas‐supported consortium: Two MAGs affiliated with *Pampinifervens* (bins 1 and 3) and a MAG (bin 2) that was most closely related to *Thermus antranikianii* (98.4% ANI; Figure 4, Table S4). The *Pampinifervens* Flu05 bin 1 MAG was 94.6% complete and comprised 51.4% of the binned community while the *Thermus* bin 2 MAG was 100% complete and comprised 35% of the binned community (Table S4). The *Pampinifervens* Flu05 bin 3 was highly incomplete (< 50%) and present in low abundance (< 15% of mapped reads (i.e., including binned and unbinned reads); Table S4) and is therefore not included in the detailed genomic analysis discussed below.

**FIGURE 6:**
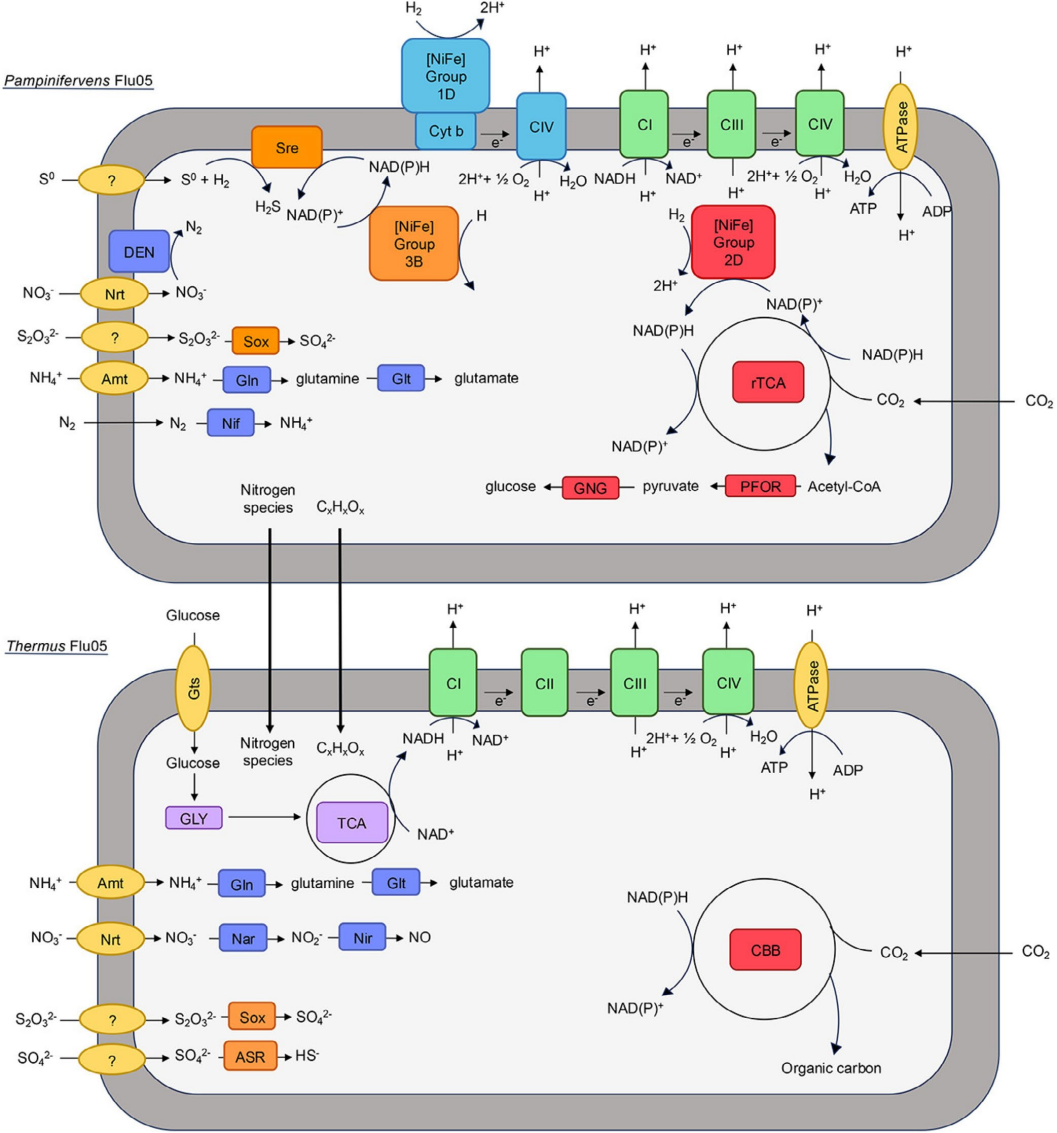
Simplified schematic of relevant metabolic capabilities of *Pampinifervens* Flu05 and *Thermus* and putative nutrient cross‐feeding between the two populations. Amt, ammonia transporter; ASR, assimilatory sulfate reduction; CI, complex 1; C II, complex 2, CIII, complex 3; CIV, complex 4 (Cox); CBB, Calvin‐Benson‐Bassham cycle (reductive pentose‐phosphate cycle); Cyt B, cytochrome B subunit; DEN, denitrification; Gln, glutamine synthetase; Glt, glutamate synthase; GLY, glycolysis; GNG, gluconeogenesis; Gts, glucose/mannose transport system; Nar, nitrate reductase; Nif, nitrogenase; [NiFe], hydrogenase; Nir, nitrite reductase; Nrt, nitrate/nitrite transporter; PFOR, pyruvate: Ferredoxin oxidoreductase; TCA cycle (citric acid cycle); rTCA, reductive TCA cycle; Sre, sulfur reductase. Proteins related to sulfur metabolism (orange), nitrogen metabolism (dark blue), carbon fixation (red), central carbon metabolism (light purple), hydrogen oxidation (light blue), and oxidative phosphorylation (green) are colour coded respectively. Pathways are depicted to only show metabolisms discussed in the text and do not show all the metabolic capabilities of the organisms described. Question marks indicate uncertainty in how those depicted metabolisms are performed.

To better understand the metabolic capabilities and potential relationships between the *Pampinifervens* Flu05 and *Thermus*, proteins encoded in the MAGs were used to develop metabolic schematics pertaining to carbon cycling, nitrogen cycling, and energy conservation. The dominant *Pampinifervens* Flu05 bin 1 MAG encoded a full rTCA cycle for CO_2_ fixation, consistent with its enrichment under autotrophic conditions and the carbon fixation pathway used by other autotrophic Aquificales (Reysenbach et al. 2009; Takacs‐Vesbach et al. 2013; Colman et al. 2024). Additionally, the MAG encodes three [NiFe]‐hydrogenase homologues, including group 1D, group 2D, and group 3B homologues. The group 1D homologue is hypothesized to be involved in H_2_ oxidation in the periplasm coupled to O_2_ respiration (Vignais et al. 2001; Peters et al. 2015) (Figure 6), consistent with the enrichment conditions that led to the consortium. The group 2D homologue is hypothesized to be cytoplasmic and involved in generation of reducing equivalents (i.e., NAD(P)H) for CO_2_ fixation (Vignais et al. 2001; Peters et al. 2015). The group 3B homologue is likely cytoplasmic and may couple with NAD(P)H (Vignais et al. 2001; Peters et al. 2015) for S^0^ reduction via sulfur reductase (Sre), which is also encoded in the MAG. *Pampinifervens* Flu05 strain encodes the Sox system for thiosulfate and S^0^ oxidation, consistent with other described Aquificales isolates (Huber et al. 1998; Eder and Huber 2002; Guiral et al. 2005; Keller et al. 2025) (Figure 6). *Pampinifervens* Flu05 also encodes a full denitrification pathway, like *Pampinifervens* isolated from China (Palmer et al. 2025). The MAG also encodes an oxidative phosphorylation pathway, including two homologues of cytochrome *c* oxidase, consistent with an ability to respire O_2_ (Figure 6).

*Pampinifervens* Flu05 encodes Nif (NifHDK) allowing for N_2_ fixation, consistent with the enrichments for this organism being provided N_2_ as their sole nitrogen source. While the *nif* operon encodes all of the structural (NifHDK) and biosynthetic genes (NifENBX) required to synthesise an active nitrogenase, it also encodes NifI_1_ and NifI_2_, proteins that post‐translationally regulate Nif in response to fixed nitrogen levels in the cell (Dodsworth et al. 2005) and that are characteristic of regulation of Nif in anaerobes (Boyd et al. 2015). Additionally, the NifK protein encoded in the Flu05 genome is split between two different open reading frames. This has previously been observed in the genome of the *Pampinifervens* isolate and *Pampinifervens* MAGs from China (Palmer et al. 2025) but has not been further investigated. Despite the gene encoding NifK being split, this organism encodes a functional nitrogenase due to its ability to grow consistently in N_2_‐fixing conditions. Low potential ferredoxin for N_2_‐fixing aerobic and autotrophic cells is typically supplied by the electron bifurcating Fix complex or via reverse ion translocation (i.e., *Rhodobacter* nitrogen fixation (RNF) complex (Poudel et al. 2018)). However, the *Pampinifervens* Flu05 MAG does not encode homologues of either Fix or RNF. It is possible that low potential Fd for Nif may be supplied by ferredoxin‐NADP^+^ reductase (Isas et al. 1995), which is encoded in the genome. Finally, the *Pampinifervens* Flu05 MAG encodes homologues of ammonia/proton antiporters that putatively facilitate transport of ammonia across the membrane and that may maintain acid–base homeostasis (Williamson et al. 2024).

The *Thermus* MAG (bin 2) encodes glycolytic, TCA, and oxidative phosphorylation pathways (Figures 5 and 6), consistent with the described ability of *Thermus* strains to grow via heterotrophic respiration with O_2_ (Chung et al. 2000). This is also consistent with preliminary data with *Thermus* isolated from the consortium (described below) that indicates an ability to grow with acetate as its sole carbon source and electron donor (data not shown). The *Thermus* MAG also encodes the Calvin‐Benson‐Bassham cycle (CBB or Calvin cycle), also known as the reductive pentose‐phosphate cycle, for CO_2_ fixation. Homologues of the Calvin cycle have previously been identified in other *Thermus* MAGs recovered from sediments across YNP (Fernandes‐Martins et al. 2023; Colman et al. 2024) (Figures 5 and 6). Consistent with the potential ability for *Thermus* to grow autotrophically, it also encodes a partial Sox pathway for thiosulfate oxidation that could be coupled to O_2_ respiration to enable energy conservation that could fuel its autotrophic metabolism. Other *Thermus* MAGs have been shown to encode Sox and often grow better in the presence of thiosulfate, even when grown heterotrophically (Chung et al. 2000; Skirnisdottir et al. 2001; Albuquerque et al. 2014). However, preliminary experiments to date have failed to demonstrate aerobic, autotrophic growth of *Thermus* with thiosulfate (data not shown). This may suggest that *Thermus* is mixotrophic with respect to electron donor and carbon source usage, features that still need to be tested. The *Thermus* MAG also encodes an N$H_{4}^{+}$ importer and enzymatic machinery to incorporate it into biomass via the glutamine synthetase/glutamate synthase (GOGAT) system. Finally, the *Thermus* MAG encodes a partial denitrification pathway, allowing for reduction of nitrate to nitric oxide, consistent with other strains of *Thermus* that have been shown to only encode genes for partial denitrification (Jiao et al. 2022; Mefferd et al. 2022).

### 3.5 Cross‐Feeding between *Pampinifervens* Flu05 and *Thermus*

#### 3.5.1 Overview

With *Pampinifervens* Flu05 and *Thermus* in a stable culture and the goal to better understand the nature of the relationship between them, growth studies were conducted to monitor the abundance of each population under both N_2_‐fixing and non‐N_2_‐fixing (N$H_{4}^{+}$‐amended) conditions with CO_2_ as the carbon source, H_2_ as the electron donor, and O_2_ as the electron acceptor. Because the three populations in the co‐culture have similar morphologies (Figure S4), an alternative approach was needed to quantify their growth dynamics. As such, primers were designed for use in qPCR assays that were specific for the 16S rRNA gene of either *Pampinifervens* Flu05 or *Thermus*. Although the subdominant *Pampinifervens* Flu05 (bin 3) MAG did not encode a 16S rRNA gene to enable primer design due to incompleteness, the primers designed for *Pampinifervens* Flu05 (bin 1) amplified the bin 3 *Pampinifervens* Flu05 as well. This was verified by isolating *Pampinifervens* Flu05 bin 3 in subsequent culture assays (verified via sequencing) and testing the primers on this strain (Figure S5). It should be noted the isolated bin 3 *Pampinifervens* genome does not encode Nif but does encode the rTCA cycle, although this could be due to the high level of incompleteness of the bin 3 *Pampinifervens* MAG (49.6% complete). Importantly, the reader is referred to a recent publication on *Pampinifervens* that discusses the spotty distribution and evolution of Nif among Aquificales genera, including recently diverged strains of *Pampinifervens* (Palmer et al. 2025).

### 3.6 Cross‐Feeding Under Non‐N_2_‐Fixing Autotrophic Conditions

**FIGURE 7:**
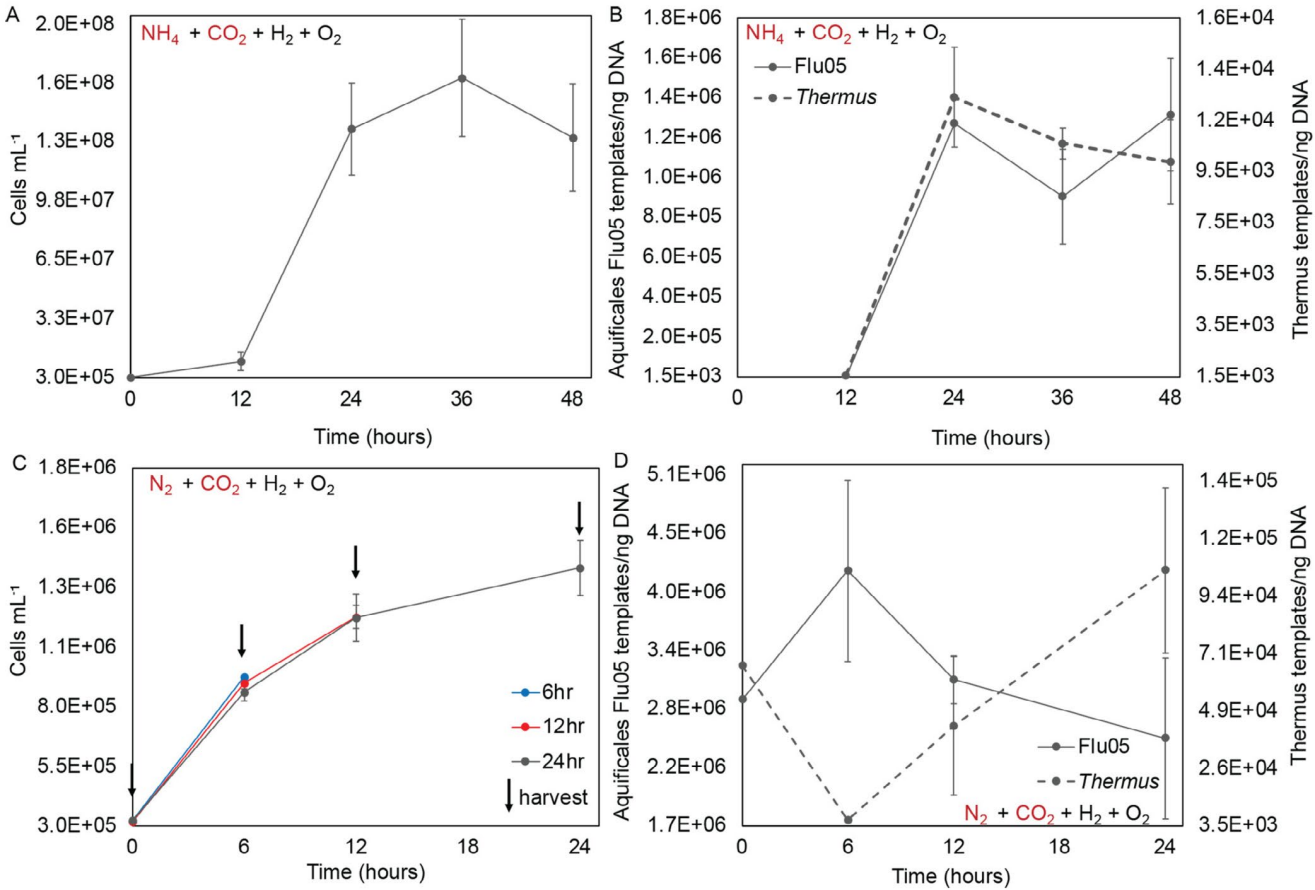
Concentration of cells and abundance of 16S rRNA gene templates in a co‐culture of *Pampinifervens* Flu05 and *Thermus* in non‐nitrogen (N_2_)‐fixing (panels A and B) and N_2_‐fixing cultivation conditions (panels C and D). 16S rRNA gene templates attributable to *Pampinifervens* Flu05 or *Thermus* were determined via quantitative PCR using primers specific to each population. Subsamples (2 mL) of non‐N_2_‐fixing cultures were sufficient for qPCR assays (panels A and B), whereas entire N_2_‐fixing cultures (55 mL) were required for qPCR assays (panels C and D). As such, triplicate cultures were harvested at 6, 12, and 24 h, as indicated by arrows in panel **C**. The composition of the headspace in non‐N_2_‐fixing cultures was 78% H_2_, 20% CO_2_, and 2% O_2_ (vol/vol) (panels A and B), and was 58% N_2_, 20% H_2_, 20% CO_2_, and 2% O_2_ (vol/vol) (panels C and D) in N_2_‐fixing conditions. All cultures were incubated on their sides at 80°C on a shaking platform incubator (50 rpm). The average and standard deviation of single measurements on triplicate cultures are shown.

To characterise growth and population dynamics between *Thermus* and *Pampinifervens* Flu05 when *Thermus* is forced to rely on *Pampinifervens* Flu05 for organic carbon, cultures were provided with N$H_{4}^{+}$ (non‐N_2_‐fixing conditions), CO_2_ as the carbon source, and H_2_/O_2_ for energy conservation. The total concentration of cells was determined via fluorescence microscopy and the concentration of *Pampinifervens* Flu05 and *Thermus* 16S rRNA gene templates was quantified via qPCR. The concentration of cells increased nearly 3 logs from 5.22 × 10^5^ to 1.66 × 10^8^ cells mL^−1^ within 36 h of incubation (Figure 7A). The cells had a short lag phase from 0 to 12 h, likely because a co‐culture grown under N_2_‐fixing conditions (not provided with N$H_{4}^{+}$) was used as the inoculum for these experiments. Due to the lag phase, the 0‐ and 6‐h timepoints were not analysed via qPCR due to the minimal changes in cell concentrations observed during this growth phase. The concentration of *Pampinifervens* Flu05 16S rRNA gene templates increased from 1.41 × 10^3^ to 1.27 × 10^6^ copies per ng of DNA between 12 and 24 h (Figure 7B) while the concentration of the *Thermus* 16S rRNA gene templates increased minimally from 1.55 × 10^3^ to 1.24 × 10^4^ copies per ng of DNA during this time. This indicates that most of the cells produced during this interval are attributed to *Pampinifervens* Flu05, although the increase in *Thermus* templates suggests successful cross‐feeding of organic carbon/electron donor. There was a minimal increase in cell concentration and *Pampinifervens* Flu05 and *Thermus* templates between 24 and 48 h, possibly indicating substrate limitation (likely O_2_ based on previous studies showing fast O_2_ drawdown in an Aquificales isolate (Keller et al. 2025)).

### 3.7 Cross‐Feeding Under N_2_‐fixing Autotrophic Conditions

To determine whether *Thermus* also relies on *Pampinifervens* Flu05 for fixed nitrogen, experiments were performed under CO_2_‐ and N_2_‐fixing conditions with a culture headspace composition of 58% N_2_, 20% CO_2_, 20% H_2_, and 2% O_2_. Production of cells in the co‐culture was far lower in N_2_‐fixing conditions when compared to non‐N_2_‐fixing conditions (Figure 7A,C), consistent with the energy and reductant demand that N_2_ fixation via Nif places on cells [minimally, 16 mol ATP and 8 low potential reducing equivalents per mol N_2_ reduced (Boyd and Peters 2013)]. Under N_2_‐fixing conditions, the concentration of cells increased from 3.0 × 10^5^ to 1.38 × 10^6^ mL^−1^ over a 24‐h incubation period (Figure 7C). Nonetheless, the cells did not experience a lag phase and increased in cell concentration within the first 6 h of incubation, only to level off after only 24 h of incubation (Figure 7C). This is likely attributable to using a co‐culture grown under N_2_‐fixing conditions as the inoculum. Because of the low cell concentrations in cultures under N_2_‐fixing conditions, a total of 16 replicates were prepared and triplicate cultures were harvested for each timepoint (0, 6, 12, and 24 h) to generate sufficient genomic DNA to permit qPCR analyses.

Between 0 and 6 h incubation, only *Pampinifervens* Flu05 16S rRNA gene templates increased slightly from 2.89 × 10^6^ to 4.16 × 10^6^ per ng of DNA (Figure 7D). Templates attributable to *Thermus* over this time decreased from 6.64 × 10^4^ to 5.78 × 10^3^ per ng of DNA, indicating that the *Pampinifervens* population is increasing in abundance within the first 6 h while the *Thermus* population is decreasing in abundance (Figure 7D). Between 6 and 24 h, templates attributable to *Pampinifervens* Flu05 decreased from 4.16 × 10^6^ to 2.51 × 10^6^ templates per ng of DNA while templates attributable to *Thermus* slowly increased from 5.77 × 10^3^ to 1.04 × 10^5^ templates per ng of DNA (Figure 7D). The dynamics of the population abundances (inferred by 16S rRNA gene templates), including the lack of a lag phase in *Pampinifervens* Flu05 and the lag phase in *Thermus* over the first 6 h of incubation, suggest that *Thermus* is dependent on the *Pampinifervens* populations to obtain fixed carbon (carbon source and electron donor) and fixed nitrogen.

Interestingly, the concentration of *Thermus* 16S rRNA gene templates reached a higher concentration in the N_2_‐fixing co‐culture than the non‐N_2_‐fixing co‐culture, reaching a maximum copy number of 1.04 × 10^5^ (±3.24 × 10^4^) templates per ng of DNA versus 1.24 × 10^4^ (±1.94 × 10^3^) templates per ng of DNA, respectively (Figure 7B,D). This is despite the total number of cells being lower in N_2_ fixing conditions. The difference in final cell template numbers is significantly different (*p* = 0.039), indicating the possibility that *Thermus* preferred the form of cross‐fed nitrogen provided by *Pampinifervens* Flu05 as opposed to ammonia that was provided in the non‐N_2_ fixing condition. It is also worth noting the substantial variation in qPCR‐based assessments of template concentration, which can likely be attributed to the way the samples were harvested for DNA extraction. Centrifugation of the co‐culture often failed to yield a solid pellet, leading to loss of biomass during aspiration of supernatant. Attempts to overcome this by filtration resulted in minimal recovery of DNA, possibly due to interference of the filter during extraction. Despite the high level of variation among replicates, the trends in the data and the difference in the maximum copy number reached by *Thermus* suggest that *Thermus* heavily relies on *Pampinifervens* under these growth conditions and prefers cross‐fed organic products of N_2_ fixation (e.g., amino acids) opposed to ammonia. While this is only one example of cross‐feeding between *Pampinifervens* and *Thermus* in culture, the extensive co‐occurrence of these two lineages suggest that these relationships may be widespread and could be taking place in the environment as well.

**FIGURE 8:**
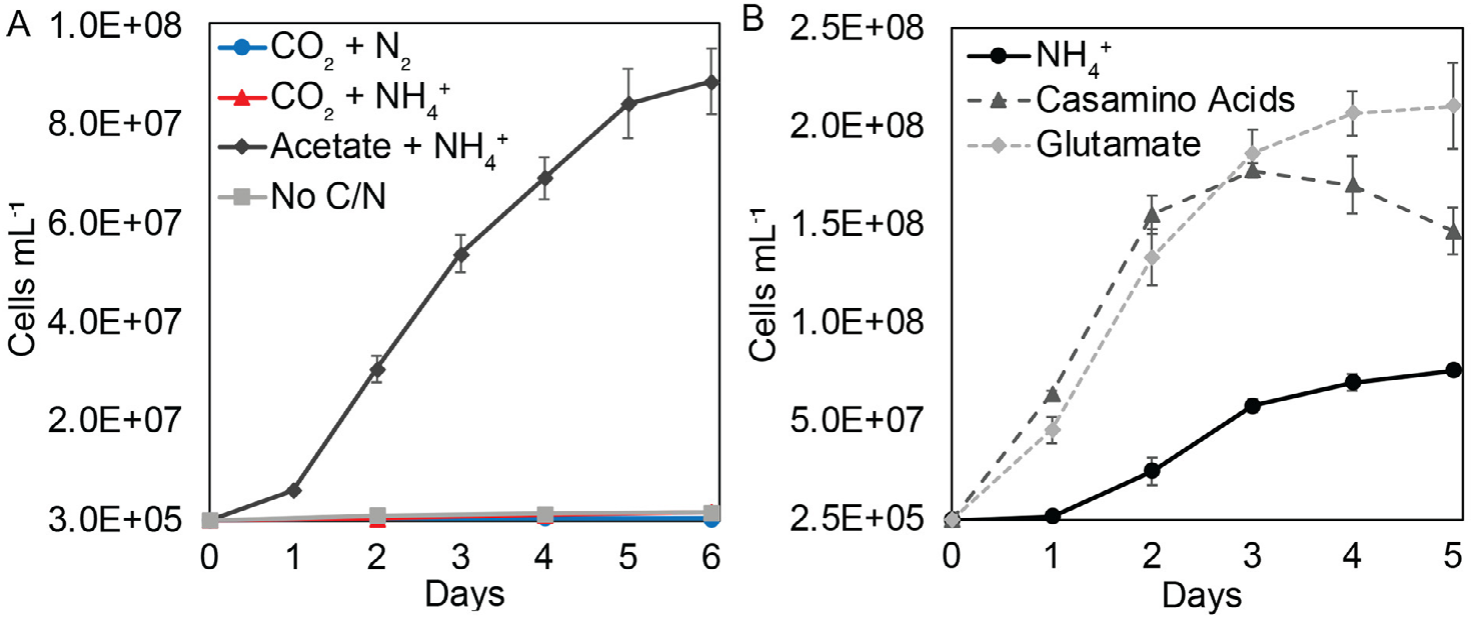
Concentration of cells in a culture of *Thermus* in isolation when grown with specified carbon and nitrogen sources. Production of *Thermus* cells when grown under carbon‐ and nitrogen‐fixing conditions (supplied as carbon dioxide (CO_2_) and dinitrogen (N_2_); blue circles) or under carbon‐fixing conditions (supplied as CO_2_ and ammonium (N$H_{4}^{+}$); red triangles) with hydrogen (H_2_) and oxygen (O_2_) provided as the electron donor and electron acceptor, respectively (panel A). The positive control *Thermus* culture was grown with acetate as its carbon source and electron donor, O_2_ as its electron acceptor, and ammonia (NH_3_/N$H_{4}^{+}$) as a nitrogen source (dark grey diamonds), while the negative control was not given a carbon or nitrogen source in an H_2_/O_2_ headspace (light grey squares). Production of *Thermus* cells when grown with 6 mM of ammonium chloride (solid black circles), casamino acids (dashed dark grey triangles), or glutamate (as glutamic acid; dashed light grey diamonds) (panel B). Cultures were provided with acetate as carbon source and electron donor and O_2_ as electron acceptor. All cultures were incubated statically at 70°C. The average and standard deviation of three replicate cultures is shown.

To further explore the dependency of *Thermus* on *Pampinifervens* Flu05 for fixed carbon and nitrogen, growth experiments were conducted with *Thermus* grown in isolation. *Thermus* was isolated from the co‐culture through a series of dilutions with acetate as the sole carbon source/electron donor and O_2_ as electron acceptor. Isolation was confirmed using primers designed for qPCR experiments and the isolated culture was then tested for its ability to grow under the conditions that have been used to maintain the co‐culture for > 2.5 years. For the experiment, the positive control was provided with acetate (5 mM) as carbon source and electron donor, O_2_ as an electron acceptor, and ammonia as the nitrogen source, while the negative control was not provided any source of carbon or nitrogen. The experimental controls were provided with CO_2_ as carbon source, H_2_ as electron donor, O_2_ as electron acceptor, and either ammonia or N_2_ as nitrogen source, the same that were used to maintain the co‐culture (Figure 8A). While *Thermus* grew well under the positive control condition, there was no measurable growth in either of the experimental conditions when compared to the negative control (Figure 8A). Slight increases seen in the two experimental conditions and the negative control can likely be attributed to carryover of organic carbon and nitrogen from the inoculum, although the inoculum was washed three times with minimal medium to limit potential carryover. These results demonstrate that *Thermus* is unable to support itself under the conditions utilised in cross‐feeding experiments and confirm that growth of *Thermus* in the co‐culture is facilitated by *Pampinifervens* Flu05 and likely involves transfer of both fixed organic carbon and nitrogen.

### 3.8 *Thermus* Growth When Provided Varying N_2_ Sources

Based on qPCR data, the abundance of *Thermus* increased more in a co‐culture growing under N_2_‐fixing conditions when compared to the co‐culture grown with N$H_{4}^{+}$, suggesting that dissolved ammonia may not be the form of fixed nitrogen that *Thermus* evolved to assimilate when growing in association with *Pampinifervens*. Based on previous research, N_2_‐fixing *Azotobacter* could cross‐feed either ammonia or amino acids to partner strains when grown in co‐culture (Wang et al. 2023), suggesting the possibility that *Pampinifervens* Flu05 could cross‐feed either ammonia or amino acids to *Thermus*. To begin to evaluate this possibility, experiments were conducted with the isolated *Thermus* strain to compare growth when cultures were provided dissolved ammonia (as ammonium chloride), glutamate (as glutamic acid), or casamino acids as nitrogen source with acetate as the source of carbon and reductant and O_2_ as electron acceptor. For results to be comparable, each nitrogen source was provided at a concentration of 6 mM (6 mM of nitrogen provided via ammonium chloride or glutamate). Although casamino acids do not have an exact nitrogen concentration, it was added at the same weight per volume percent as glutamic acid. The concentration of cells in cultures provided with glutamate or casamino acids as the nitrogen source increased significantly faster compared to the ammonia‐grown cells and reached a maximum cell concentration of 1.8 × 10^8^ and 2.1 × 10^8^ cells mL^−1^ respectively, when compared to 7.7 × 10^7^ cells mL^−1^ in ammonia‐grown cultures (Figure 8B). These data indicate that *Thermus* grows better on amino acids as a nitrogen source (and possibly as a carbon source) and may help explain better growth of *Thermus* in co‐culture with *Pampinifervens* Flu05 under N_2_‐fixing conditions.

## 4 Conclusions

The strong pattern in the co‐occurrence of Aquificales and *Thermus* in YNP and Iceland hot springs suggested possible interactions between these organisms, possibly at the level of nutrient cross‐feeding. While DOC and dissolved ammonia were low in both YNP and Iceland hot springs, they were significantly lower in Iceland, pointing to the potential for enrichment in autotrophic and diazotrophic metabolisms in Iceland and an increased need for cross‐feeding of both autochthonous fixed carbon and nitrogen to support heterotrophic populations. Potentially consistent with this hypothesis, putatively diazotrophic Aquificales populations were more widespread and abundant in Iceland than YNP hot springs, while the abundance of autotrophic Aquificales populations was similar between the two regions.

To begin to understand the nature of potential interactions between Aquificales and *Thermus*, a co‐culture containing *Pampinifervens* and *Thermus* was produced from an Icelandic hot spring. Growth experiments indicated that *Thermus* was dependent on *Pampinifervens* for both fixed carbon and nitrogen under the culture conditions used in the study. This co‐culture has been maintained in a laboratory environment with repeated transfers for > 2.5 years, further suggesting cross‐feeding of carbon and nitrogen allows the *Thermus* to survive in nutrient‐limited conditions. Surprisingly, *Thermus* grew better under conditions that required *Pampinifervens* to fix both carbon and nitrogen when compared to conditions that required *Pampinifervens* to fix carbon alone. Additional growth studies with *Thermus* in isolation showed that it grew faster and to a higher cell density when provided with glutamate or casamino acids as nitrogen source (and possible carbon source) versus dissolved ammonia, supporting the results observed in the cross‐feeding experiments.

While there is precedent for amino acids (e.g., glutamate) as being metabolites that are cross‐fed between diazotrophic bacteria and other bacteria, it is unknown if this is the true metabolite that is exchanged in this co‐culture. Exometabolomics would be the preferred method to determine the identity of the nitrogenase compound (and organic carbon substrate) that *Pampinifervens* is providing to the *Thermus* population, though it is unlikely that it would accumulate to detectable levels in the co‐culture due to rapid consumption by *Thermus*. While it was possible to isolate *Thermus* and the less abundant *Pampinifervens* (bin 3) from the co‐culture, repeated attempts to isolate the dominant Aquificales population (bin 1) for use in an exometabolomics analysis were unsuccessful. Importantly, the less abundant *Pampinifervens* population does not encode Nif and therefore could not be used to perform such experiments. The fact that it was not possible to separate the dominant *Pampinifervens* (bin 1) from *Thermus* suggests the possibility that *Thermus* is providing metabolites to *Pampinifervens*, and that this relationship is enhanced under N_2_‐fixing and autotrophic growth conditions. Based on genomic data, growth studies conducted herein, and preceding studies investigating cross‐feeding in a diazotrophic co‐culture (Wang et al. 2023), possible metabolites being cross‐fed to *Thermus* could include but are not limited to acetate, glucose, and amino acids, such as glutamate and glutamine (Wang et al. 2023). Additional studies are required to identify potential metabolites cross‐fed from *Thermus* to *Pampinifervens*.

Aquificales are often the dominant autotrophic population in high temperature hot springs globally, and in many cases encode for N_2_ fixation potential. As such, it is likely that this group of organisms would be responsible for supplying fixed carbon (and possibly fixed nitrogen) to sustain other members of hot spring communities. Given that *Thermus* is one of many taxa that likely are supplied with fixed carbon and/or nitrogen by Aquificales in hot springs, it is suggested that Aquificales have a disproportionate, possibly keystone, role within their microbial communities, and their removal from such communities would have a cascading negative impact on ecosystem function. This is particularly true since most of the metagenomes analysed here and that showed a strong pattern in the co‐distribution of Aquificales and *Thermus* are from the sediments of hot springs. Aquificales are more dominant in the water column of hot springs (Hou et al. 2013; Colman et al. 2016; Fernandes‐Martins et al. 2021; Fernandes‐Martins et al. 2023; Keller et al. 2023), so what is described here is likely an underestimate of the true importance of Aquificales in the provision of fixed carbon and/or nitrogen to other members of the community. Future studies are needed to determine how members within the order Aquificales interact with and shape hot spring microbial communities, both in sediments and in the water column.

## Author Contributions

**Lisa M. Keller:** conceptualization, investigation, writing – original draft, methodology, validation, visualization, writing – review and editing, formal analysis, data curation. **Daniel R. Colman:** conceptualization, funding acquisition, writing – review and editing, formal analysis, supervision, investigation, software. **Andri Stefánsson:** investigation, writing – review and editing, data curation, resources. **Eric S. Boyd:** conceptualization, investigation, funding acquisition, writing – original draft, writing – review and editing, visualization, validation, methodology, formal analysis, project administration, supervision, resources.

## Funding

This work was supported by the National Aeronautics and Space Administration (80NSSC19M0150) and W. M. Keck Foundation (MSU D19).

## Conflicts of Interest

The authors declare no conflicts of interest.

## Supporting information


**Data S1:** emi70225‐sup‐0001‐Tables.xlsx.

**Data S2:** emi70225‐sup‐0002‐Figures.docx.

## Data Availability

All data generated or analysed during this study are included in the text and figures of this published article and its Supporting Information. Sequencing data is publicly available through NCBI under BioProject number PRJNA1255289.
