# Supplementary material for: Cross‐Feeding of Carbon and Nitrogen Between Aquificales and Thermus in Hot Springs
Source: Environ Microbiol. 2026 Jan 7;28(1):e70225. doi: 10.1111/1462-2920.70225 (PMC12780486; doi:10.1111/1462-2920.70225)
Supplement: Supplementary file 2 — Data S2: emi70225‐sup‐0002‐Figures.docx. [file EMI-28-e70225-s001.docx]

**Supplemental figures:**

**Supplemental Figure 1. The distribution and relative abundance of Aquificales- and *Thermus*-affiliated metagenome-assembled genomes (MAGs) recovered from 105 Yellowstone and Iceland hot spring microbial communities separated into individual plots for each genus**. Each bubble represents a MAG affiliated with a member of the order Aquificales or genus *Thermus* and is plotted as a function of the pH and temperature of the hot spring that it was recovered from. The size of the bubble corresponds to the relative abundance of that MAG (% of binned reads) in the hot spring sediment community and the color corresponds to the genus. Each panel depicts an individual genus shown in Fig. 2.

**Supplemental Figure 2. Phylogenomic reconstruction of members within the genus *Thermus*.** This figure corresponds with figure 4 in the main text and is showing the uncollapsed phylogeny of *Thermus* affiliated metagenome assembled genomes (MAGs). Names with the asterisk indicate MAGs that were generated from Yellowstone National Park (YNP) and Iceland that were analyzed throughout the maintext. Bolded MAGs indicate the encoded ability to fix CO_2_ (Supplemental Table 6).

**
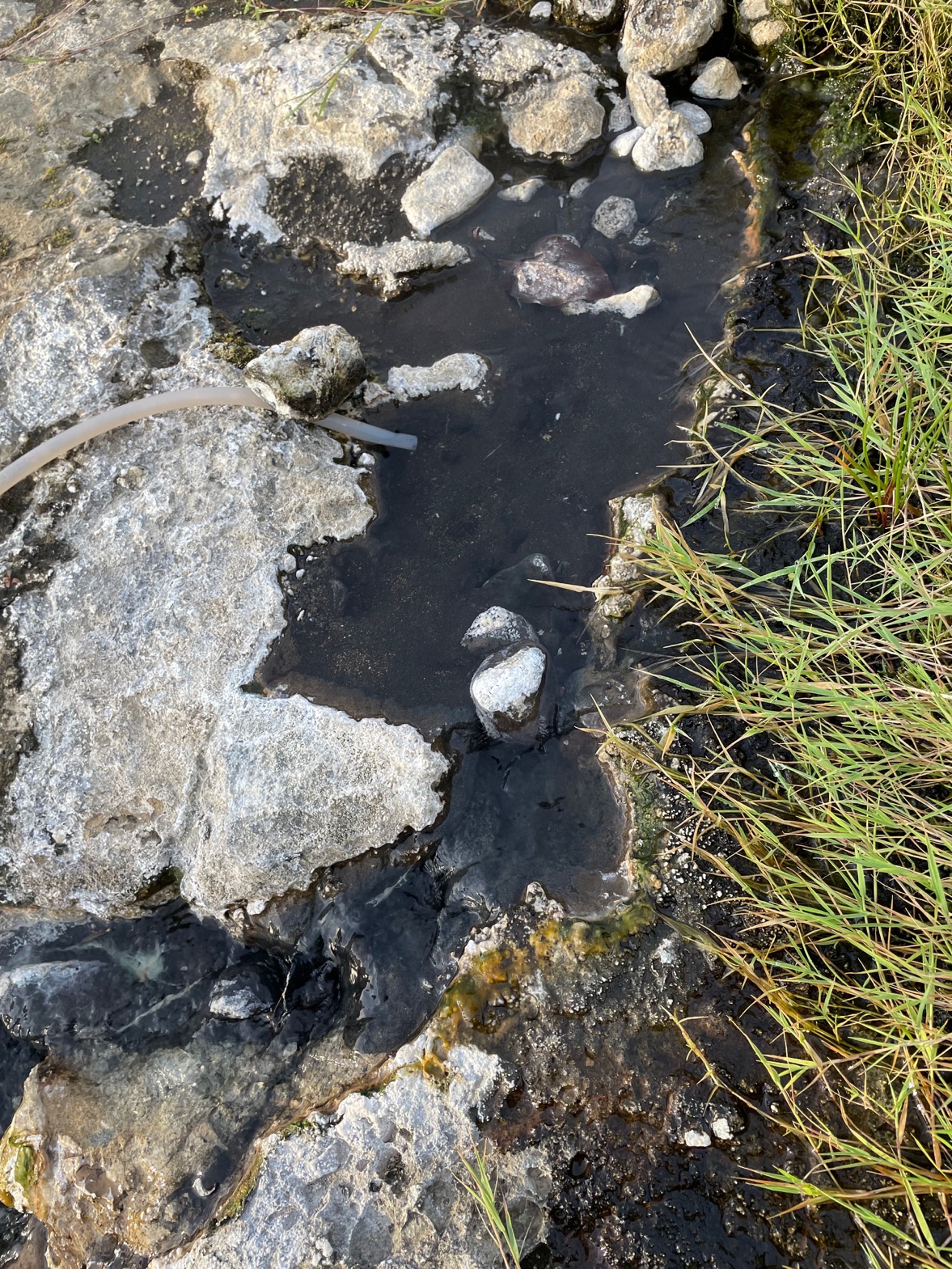
**

**Supplemental Figure 3. Flúðir 5 (Flu05) hot spring located in Flúðir, Iceland.** Red arrow indicates the location in Flu05 where sediments and water were collected for culturing. The blue arrow is showing the white streamers that were in the outflow channel of the spring. All geochemical measurements reported in the manuscript were performed *in situ* at the location depicted by the red arrow or on samples collected from that location.

**
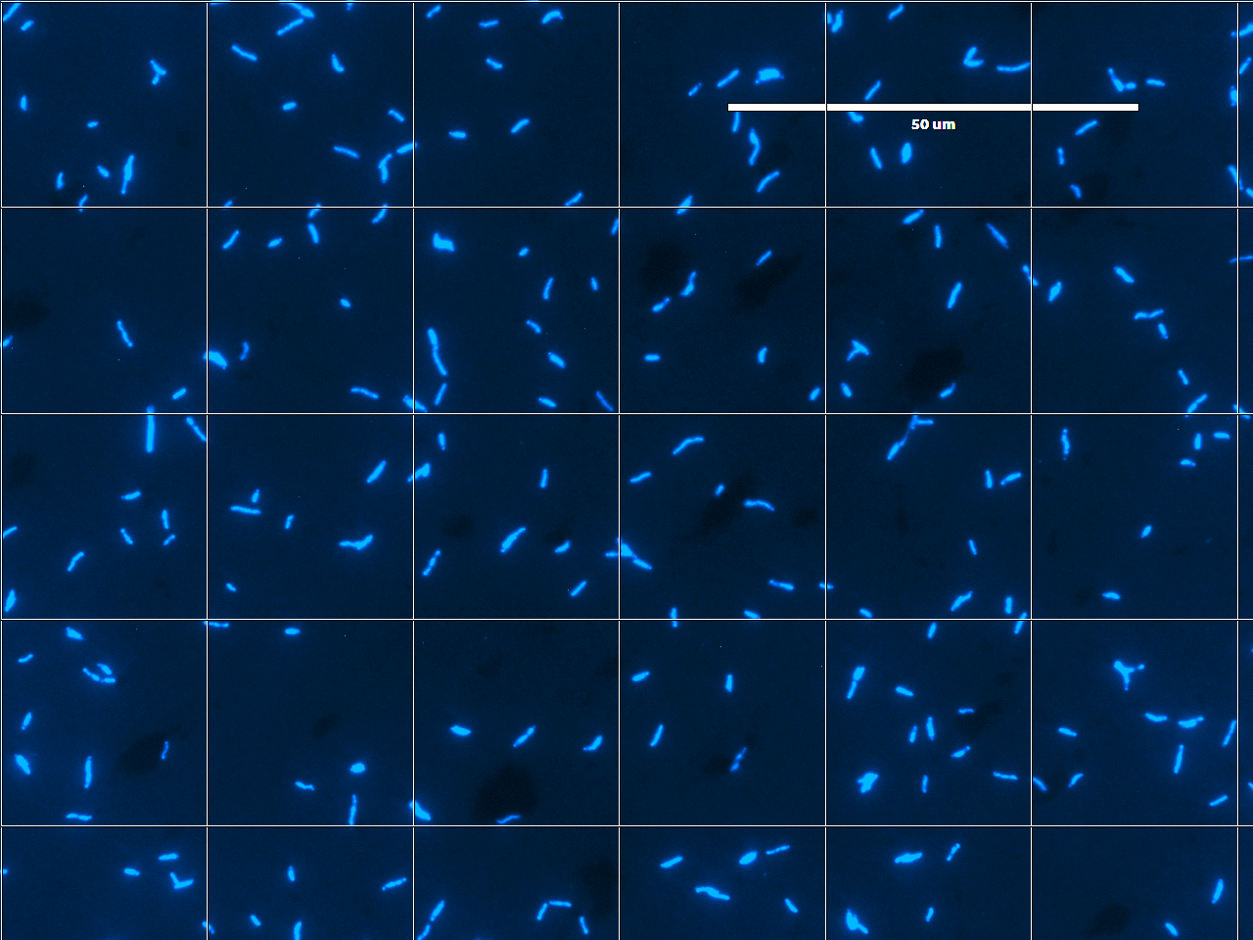
**

**Supplemental Figure 4. Fluorescent microscopic image of cells in the *Pampinifervens* Flu05 and *Thermus* co-culture growing under carbon dioxide (CO_2_)-fixing and nitrogen (N_2_)-fixing conditions.** DAPI-stained cells were imaged with a 60X objective. The scale bar is 50 µm.

**Supplemental Figure 5. Optimization of PCR primers designed to amplify the 16S rRNA gene region of *Pampinifervens* Flu05 or *Thermus* as a proxy for growth in co-cultures.**
